# Impact of Acute and Chronic Cannabis Use on Stress Response Regulation: Challenging the Belief That Cannabis Is an Effective Method for Coping

**DOI:** 10.3389/fpsyg.2021.687106

**Published:** 2021-07-01

**Authors:** Mustafa al'Absi, Alicia M. Allen

**Affiliations:** ^1^Department of Family Medicine and Biobehavioral Health, University of Minnesota Medical School, Duluth, MN, United States; ^2^Department of Family and Community Medicine, University of Arizona, Tucson, AZ, United States

**Keywords:** stress, cannabis, emotions, early life adversities, addiction, coping

## Abstract

Although research has only recently started to examine the impact of cannabis use on stress response, there is some evidence that indicates acute and chronic impacts of cannabis on these processes. In this paper, we review processes involved in regulating the stress response and we review the influence of acute and chronic exposure to cannabis on patterns and regulation of the stress response. We also highlight the role of stress as a risk factor for initiation and maintenance of cannabis use. In this context, we examine moderating variables, including sex and life adversity. In light of recent observations indicating increasing prevalence of cannabis use during pregnancy, we provide additional focus on cannabis use in this vulnerable population, including how acute and chronic stress may predispose some individuals to use cannabis during pregnancy. While this line of research is in its infancy, we review available articles that focus on the perinatal period and that examined the association between cannabis use and various life stressors, including partner violence, job loss, and lack of housing. We also review psychiatric co-morbidities (e.g., post-traumatic stress disorder, anxiety). A better understanding of the way stress and cannabis use relate within the general population, as well as within certain subgroups that may be at a greater risk of using and/or at greater risk for adverse outcomes of use, may lead to the development of novel prevention and intervention approaches.

## Introduction

Cannabis is the most widely used psychoactive substance in the world (UNODC, 2020). Changes in the legal and regulatory status of the substance have contributed to escalation of use in recent years, with a growing number of countries and regions legalizing and decriminalizing cannabis for medical and recreational purposes (Leweke and Koethe, 2008; Office, 2012; Peters et al., 2012; Administration SAaMHS, 2013; Lev-Ran et al., 2013; Hurd et al., 2014; Ahrnsbrak et al., 2016; Carliner et al., 2017; Steigerwald et al., 2018). Stress is a well-known risk factor for all stages of drug use–from initiation to maintenance and dependence to relapse–for many substances, including cannabis (al'Absi et al., 2004, 2007; Hyman and Sinha, 2009). Preliminary evidence shows that chronic cannabis use may alter stress responses (Cuttler et al., 2017; DeAngelis and al'Absi, 2020). Moreover, there are documented sex differences and pregnancy-specific risks for substance use, including cannabis (Hernandez-Avila et al., 2004; Conner et al., 2016; Gunn et al., 2016; Campbell et al., 2018; Serino Ma et al., 2018; Brown et al., 2019b; Nashed et al., 2020; Sarrafpour et al., 2020). Consequently, the roles of sex and pregnancy should be considered when describing the relationship between stress and cannabis use.

In this paper, we discuss the acute and chronic effects of cannabis, stress, and their effects on various physiological systems. We also discuss mechanisms that may mediate effects of stress and cannabis use and that may increase risk for long-term dependence. Additionally, we examine sex as a moderator of these relationships, and we examine the impacts of cannabis and stress during pregnancy. To inform our discussion, we reviewed titles and abstracts to identify relevant articles that matched searches in Pubmed, Embase, Scopus, and PsychINFo using multiple combinations of the following terms: “stress response,” “cortisol,” “cardiovascular reactivity,” or “hypothalamic pituitary adrenal (HPA) axis” and each of the following terms: “endocannabinoids,” “tetrahydrocannabinol (THC)”, “cannabis,” or “marijuana,” as well as “pregnancy,” “prenatal,” “post-partum,” and “perinatal.” Overall, this review will highlight how cannabis use is linked to stress response dysregulation, and it will challenge the belief that cannabis is an effective coping method when dealing with stress.

## Acute Effects of Cannabis

The two primary chemical constituents of cannabis are THC and cannabidiol (CBD). THC is the main psychoactive ingredient in cannabis, and both THC and CBD bind to cannabinoid CB1 and CB2 receptors (Pertwee, 2008a,b). While CB2 receptors are predominantly in immune cells and play a role in the anti-nociceptive effects of cannabis, CB1 receptors are located in central and peripheral neurons. CB1 receptors are located in the amygdala, hypothalamus, hippocampus, neocortex, and basal ganglia (Pertwee, 2008a,b). These receptors may, therefore, mediate the influence of cannabis on emotion regulation and stress responses.

The acute effects of THC include feeling a “high” associated with mood changes, alterations in the sense of time, experiencing difficulty in thinking clearly, and alterations in processing sensory information from surrounding stimuli. These effects tend to occur within 30–60 min after consumption of cannabis. In contrast, CBD tends to produce acute reductions in tension and anxiety. For example, research has focused on the potential effects of CBD in anxiety-related symptoms and post-traumatic stress disorder (PTSD) focusing on CBD potential therapeutic effects (Blessing et al., 2015; Steenkamp et al., 2017). In this context, research in animal models demonstrates potential effects of CBD on anxiety-related memory processes (Prud'homme et al., 2015; Jurkus et al., 2016) and acute effects of CBD seems to occur through learned fear and impairing the ability to acquire fear conditioning in preclinical models (Levin et al., 2012; Jurkus et al., 2016).

## Acute Stress Response

Psychological stress activates several biological systems, including the HPA axis and the autonomic nervous system (Chrousos and Gold, 1992; McEwen, 2007; Kudielka and Wüst, 2010). The HPA axis, through release of adrenocorticotropic hormone (ACTH) and cortisol, performs a central role in directing responses to stress and plays an essential role in mediating stress effects on various brain functions (McEwen et al., 2015, 2016). In addition to its effects on the HPA axis, psychological stress also activates other systems, including the sympathetic nervous system (Mills and Dimsdale, 1992; al'Absi and Arnett, 2000; Murison, 2016), which evolutionarily acts to generate energy to fight or flight during exposure to stress.

## Effects of Acute Cannabis Use on Stress Responses

It has long been known that acute administration of THC to a naïve subject, via either smoking or controlled intravenous doses, activates stress-response systems (Cone et al., 1986; Ranganathan et al., 2009; Kleinloog et al., 2012; Klumpers et al., 2012; Cservenka et al., 2018). For instance, acute THC via smoking increases heart rate (Naliboff et al., 1976). In addition, acute administration of THC via a smoked cannabis cigarette results in an increase in cortisol within 15 min that is sustained for 75 min (Cone et al., 1986). It should be noted, however, that this stimulatory effect has not been consistently observed (Dax et al., 1989; Childs et al., 2017). There is also evidence of tolerance developing for the HPA response, as evidence by reduced cortisol response to cannabis use (Murphy et al., 1998). Tolerance has also been found for other outcomes, including behavioral, subjective, and cognitive responses to cannabis (D'Souza et al., 2004, 2008; Ranganathan et al., 2009), which is consistent with preclinical evidence of tolerance (Murphy et al., 1998; González et al., 2005; Pagotto et al., 2006).

## Effects of Chronic Cannabis Use on Stress Responses

Consistent with findings related to tobacco and alcohol (Lovallo et al., 2000; Sorocco et al., 2006; al'Absi, 2018; al'Absi et al., 2020), there is evidence that chronically using cannabis (for a year or longer) may also be associated with blunted stress responses (Benowitz et al., 1976; Buckner et al., 2006; Ranganathan et al., 2009; McRae-Clark et al., 2011; Somaini et al., 2012; Cuttler et al., 2017; Cservenka et al., 2018; DeAngelis and al'Absi, 2020). We also note studies have shown increases in ACTH responses to stress among individuals who use cannabis (McRae-Clark et al., 2011; Fox et al., 2013). However, this literature is less developed and little has been done to fully characterize the impact of chronic cannabis use on human adrenocortical function, particularly the diurnal curve and systematically examine the impact of withdrawal and dysregulation associated with risk for maintenance of use. While some have conducted well-controlled experimental stress studies, many investigations did not include controls who did not use cannabis (McRae-Clark et al., 2011, 2013) or they relied heavily on reviews of animal studies (Enoch, 2011). Still others have focused exclusively on behavioral or psychological factors (Hyman and Sinha, 2009). Frequent use of cannabis (>5 days/week for at least 1 year) appears to be associated with higher basal cortisol (King et al., 2011) and a blunted cortisol reaction to acute THC administration (Benowitz et al., 1976; Ranganathan et al., 2009). Similarly, compared to those who do not use cannabis, those who use demonstrate an attenuated cortisol response to negative emotions (Somaini et al., 2012). Both individuals who report early- and late-onset of cannabis use demonstrate a relatively flattened diurnal curve with a smaller awakening response tended to predict early onset of use (Huizink et al., 2006). This effect appears to be mediated by cannabinoid CB1 receptor function, since blockade of this receptor in participants has been shown to disrupt plasma cortisol responses, although cortisol and THC concentrations are inversely correlated (Goodwin et al., 2012).

There is also evidence that chronic stress may influence endocannabinoids (eCB) indicating reduction in quantity of CB1 receptors; an effect that may be mediated by activation of glucocorticoid receptors (Dlugos et al., 2012; Morena et al., 2016; Balsevich et al., 2017). There may be beneficial effects of eCBs on stress responses and the impact of chronic stress occur through regulation of glucocorticoid release. As such, eCBs may be considered an important element in promoting resilience (Hill et al., 2010a,b; Russo et al., 2012). For example, there is evidence that disruption of the eCB system is associated with poor adaptation to stress and that a deficient eCB system is implicated in psychopathology and emotional disorders (Parolaro et al., 2010; Marco and Laviola, 2012; Tan et al., 2014; Boorman et al., 2016; Ney et al., 2018). On the other hand, blocking CB1 enhances activation of neurons within the PVN and it enhances HPA activity, leading to increased stress responses (Pi-Sunyer et al., 2006; Gorzalka et al., 2008). Related to emotion regulation deficits, there is also evidence that deficient eCB systems may increase risk of developing PTSD and major depressive disorder (MDD) (Bluett et al., 2017; Worley et al., 2018).

There are parameters that may modify these observed relationships, including length and levels of use of cannabis. As an example, daily cannabis use may be associated with higher salivary cortisol compared to those who do not use (King et al., 2011), although no specific examination of the stress response was undertaken in this study. Yet, in another study, there was no difference in stress-related cortisol and dopamine systems analysis via positron emission tomography (PET) scans of chronic users of cannabis (Mizrahi et al., 2013). We note, however, inconsistent findings may be due to diverse and varying methodological approaches to assess effects of cannabis, including the different types of measures and specimens (e.g., blood vs. saliva), diurnal timing of the sampling (e.g., morning vs. evening), timing in relation to cannabis use (e.g., before vs. after use), as well as with the specific populations studied (e.g., among those who use cannabis only vs. poly-substance users vs. individuals who do not use or participants with mental health conditions).

## Acute and Chronic Stress As Risk Factors For Cannabis Use

The impact of stress on substance use has been demonstrated in two areas, initiation and maintenance of use, including relapse. For example, stress and early life adversity predict the onset and use patterns of multiple drugs of abuse, including cannabis (Sinha et al., 2011; Fox et al., 2013; Heron et al., 2013; van der Pol et al., 2013; Myers et al., 2014). Research has also shown an association between negative affect and cannabis use (Shrier et al., 2014, 2018; Buckner et al., 2015). These relationships may be enhanced in the presence of other triggers, such as the acute stress may increase craving for cannabis among chronic users in the presence of cannabis-related cues (McRae-Clark et al., 2011). Additionally, research on the influence of the role of stress responses in predicting cannabis use has also been undertaken in the context of longitudinal studies of adolescents and young adults. In one study, blunted cortisol stress response to a laboratory social was associated with an increase in risk of lifetime cannabis use as well as the repeated current cannabis use (van Leeuwen et al., 2011).

## Sex Differences in The Relationships Between Cannabis Use and Stress

Males are more likely to report current cannabis use compared to females (SAMSHA CfBHSaQ, 2016), but the progression from onset of regular cannabis use to dependence is steeper in females relative to males (Hernandez-Avila et al., 2004). This sex difference may be driven by sex-specific effects of cannabis. Animal studies found that THC was metabolized to its highly potent compounds in female rats while it was metabolized to various compounds in male rats (Narimatsu et al., 1991). Female adult rats, but not male adult rats, showed symptoms analogous to depression when chronically treated with THC during adolescence (Rubino et al., 2008). Further, in human studies, females who use cannabis had more craving for cannabis (King et al., 2011) and more physical withdrawal symptoms (Heishman et al., 2009; Copersino et al., 2010; Levin et al., 2010) than males who use cannabis. However, in research focused on testing acute effects of cannabis (or THC), inconsistent findings have been observed, with some indicating that females were more likely to report dizziness than males, while other studies showed that males reported more subjective effects than females (Penetar et al., 2005; Haney, 2007; Sholler et al., 2020).

Additionally, there may be sex differences in the association between stress and cannabis use (van Leeuwen et al., 2011; Fattore, 2013; Chao et al., 2018; Farquhar et al., 2019; Matheson et al., 2020). This is important because stress reduction is commonly cited as a motive for cannabis use (Copeland et al., 2001). One recent study (King et al., 2011) examined changes in brain function and hormonal activity associated with neuropsychological tests among those who use cannabis compared to those who did not. The results indicated a significant positive correlation between the superior frontal gyrus activation and cortisol concentration in female who did not use cannabis; however, an inverse relationship was found in females who use cannabis. Interestingly, no correlation was found in males who use cannabis nor in males and females who did not use cannabis (King et al., 2011).

One contributor to sex differences in the cannabis-stress link may be ovarian hormones (Greenfield et al., 2010; Fattore, 2013; Antinori and Fattore, 2017; Cooper and Craft, 2018; Ney et al., 2018; Raymundi et al., 2020). Cannabinoids influence the hypothalamic–pituitary–gonadal (HPG) axis (López et al., 2010; Craft et al., 2013; Marusich et al., 2014; Wakley et al., 2014). Based on animal models, the density of endocannabinoid receptors in the brain, including hypothalamus and pituitary, change across the estrous cycle (González et al., 2000, 2005; López et al., 2010), suggesting that neuropsychological impacts of cannabis use may differ across the human menstrual cycle. Ovarian hormones impact drug-taking behaviors for a variety of substances, including cannabis (Lynch et al., 2002; Carroll and Anker, 2010; Becker and Koob, 2016). For example, preclinical research indicates that female rats with intact ovaries had heightened self-administration, cue-induced reinstatement behavior, and drug-induced reinstatement behavior in response to WIN 55,212-2 (a drug used instead of THC for intravenious animal studies) as compared to female rats who were ovarectomized and male rats (Fattore et al., 2007, 2010); suggesting that estradiol may reinforce the effects of cannabis (Brents, 2016). In clinical literature, depressed mood increased the use of cannabis in women with premenstrual dysphoric disorder (PMDD) differentially across the menstrual cycle; suggesting both ovarian hormones and adverse moods may influence cannabis use in those in a chronically stressed state (Joyce et al., 2021). Indeed, research has begun to explore progesterone as a treatment for cannabis use disorder (Sherman et al., 2019).

Research examining sex and ovarian hormones (e.g., progesterone, estradiol) in the effects of cannabis use has been limited largely due to methodological issues, such as limited sample size, a lack of diversity within samples, and limited assessment (e.g., no measurement of ovarian hormones). Most studies included males only or included both sexes but controlled for its variance (e.g., using covariate to control for sex instead of examining it directly). An additional limitation in this literature includes a lack of focus on gender. While stress response is a biological phenomenon, it can be differentially influenced by societal factors such as gender identity (e.g., Motta-Mena and Puts, 2017; Juster et al., 2019; Passarelli et al., 2021) and gender discrimination (e.g., Huynh et al., 2016; Volpe et al., 2020). However, the above reviewed studies suggest its importance. These findings generally suggest that females have greater sensitivity to reinforcing and subjective effects of cannabinoids than males (Craft, 2005; Craft et al., 2013; Nia et al., 2018). Taken together, findings from human and animal studies suggest the importance of additional research to disentangle potential sex-specific effects on the relationship between cannabis use and stress, as well as determining sex differences in long-term impacts of cannabis use.

## Stress and Cannabis Use During Pregnancy

While there has been some conflict regarding the health effects of cannabis use during pregnancy, the literature is now converging to indicate that cannabis use during pregnancy is harmful, as it is linked to low birth weight, preterm birth, and admission to neonatal intensive care (Conner et al., 2016; Gunn et al., 2016; Campbell et al., 2018; Serino Ma et al., 2018; Brown et al., 2019b; Nashed et al., 2020; Sarrafpour et al., 2020). This emergence of literature may be related to the fact that the potency of THC in cannabis has increased by 375% from the 1980s to 2015 (ElSohly et al., 2016; Brown et al., 2019b). THC readily crosses the placental barrier and has a variety of short- and long-term effects on the developing fetus. In addition to adverse birth outcomes, in utero exposure to cannabis in preclinical models has been linked to anxiety-like behavior in adolescents and adults as well as to dose-dependent reductions in dopamine receptors (Nashed et al., 2020). Additionally, in clinical studies, in utero exposure to cannabis has been shown to adversely affect sleep in infants, toddlers, and pre-teens; it has also been linked to attenuated cortisol responses to stressors in both infants and kindergarteners (Nashed et al., 2020). Cannabis post-pregnancy also appears to suppress lactation, perhaps via the suppression of prolactin and/or oxytocin as observed in rodent models (Brents, 2016).

Despite the growing evidence for harmful effects of cannabis use during pregnancy, many pregnant women who report using cannabis also report low perceptions of harm (Sarrafpour et al., 2020). Specifically, 70% of pregnant women reported perceiving that there was “no risk” or “slight risk” if women use cannabis 1–2 times per week during pregnancy (Ko et al., 2018; Corsi et al., 2020). Perceived risk has declined over the past two decades, while both the acceptance and prevalence of use by pregnant women has increased (Ashford et al., 2019). Among pregnant women who have used cannabis in the past 30 days, in 2005 25.8% of them reported “no risk” related to in utero cannabis exposure whereas in 2015, 65.4% reported the same (Jarlenski et al., 2017). In a qualitative study, Chang and colleagues found that pregnant women reported a belief that cannabis was natural and safe because it was a plant; pregnant women also reported conflicting opinions about the addictiveness of cannabis (Chang et al., 2019).

Like the general population, one of the most common reasons pregnant women report using cannabis during pregnancy is to manage stress, to calm down, or to relax (Ko et al., 2018; Chang et al., 2019). Indeed, cannabis use during pregnancy is more common in women with anxiety disorders, history of trauma, and symptoms of stress (Young-Wolff et al., 2020). Our prior research indicates that women who experienced a stressful life event (e.g., divorce, job loss) in the year prior to childbirth were at higher odds for reporting cannabis use during pregnancy (Allen et al., 2020). Similarly, past year sexual intimate partner violence placed women at more than 2 times higher odds of cannabis use during pregnancy (Bacchus et al., 2018). Further, a prospective examination of the relationship between symptoms of PTSD and cannabis use in pregnant women using ecological momentary assessments found that peak PTSD symptoms were significantly and temporally related to cannabis use (Sanjuan et al., 2019). In contrast, however, a cross-sectional examination of perceived stress scores with self-reported cannabis use during the first trimester indicated a null relationship (Ashford et al., 2019), perhaps due to overall low perceived stress scores, lack of variability, or reliance on self-reported use. Similarly, Ellis and colleagues (Ellis et al., 2019) did not observe a link between psychological distress and cannabis use during pregnancy among justice-involved women. Together, these three studies (Ashford et al., 2019; Ellis et al., 2019; Sanjuan et al., 2019) suggest that the relationship between subjective stress and cannabis use may be complicated with daily fluctuation that may not be detectable via onetime assessments. Numerous demographic variables (e.g., younger age, lower education, lower income), use of other substances (e.g., nicotine, alcohol) during pregnancy, and depression are known predictors of cannabis use during pregnancy. Despite the increasing rates of cannabis use during pregnancy (Brown et al., 2019b), there is currently no published literature on links between cannabis use and physiological markers of stress (e.g., cortisol and eCB) during pregnancy. This is important missing information, given numerous known changes to physiological stress responses during pregnancy (La Marca-Ghaemmaghami et al., 2015).

## Discussion

Stress and cannabis use likely have a bidirectional relationship, such that stress likely promotes and maintains cannabis use, while cannabis use likely alters stress responses both acutely and chronically in ways that may, ultimately in the long-term, increase perceived stress and risk for anxiety and depression (Rubino et al., 2008; Hyman and Sinha, 2009; Delforterie et al., 2015; Blanco et al., 2016; Borges et al., 2016; Danielsson et al., 2016; Feingold et al., 2016; Di Forti et al., 2019; Gobbi et al., 2019; Hosseini and Oremus, 2019; Scherma et al., 2020; Xue et al., 2021). This indicates that cannabis, deceptive perceptions to the contrary, may not be an effective coping mechanism for stress. While the literature focusing on impacts of stress on factors that influence risk for cannabis use remains in its early stage, there is a growing literature on the impact of chronic cannabis use on stress responses, including emotion regulation processes during acute stress. The literature reviewed here indicates blunted emotion regulation and occasional observations of blunted physiological responses associated with regular cannabis use (Li et al., 2005; Somaini et al., 2012; Cuttler et al., 2017).

While the research around adverse effects of cannabis has not been fully investigated, it is worth noting here that the effects of cannabis that interrupt observed blunted stress responses with cannabis uses as a potential advantage (e.g., Cuttler et al., 2017; DeAngelis and al'Absi, 2020). This side of the discussion is based on findings suggesting that cannabis or THC is associated with reduced responses to affective and threatening stimuli (Phan et al., 2008; Gruber et al., 2009; Cornelius et al., 2010; Gorka et al., 2014; Conrad et al., 2015; Childs et al., 2017; Crane and Phan, 2021). In the context of acute administration, these effects seem to be dose-specific and more likely to occur with lower doses of THC and to subjective rather than physiological response to stress (Childs et al., 2017). This perspective of advantageous blunting of stress responding attributable to cannabis use has also been proposed in the context of cortisol responses to stress (Cuttler et al., 2017; Chao et al., 2018). It is, nevertheless, premature to draw any conclusion about the potential benefit or detriment of cannabis use.

More specifically relevant to this review is our observation of differences in sex-specific patterns and susceptibility to the impact of stress in patterns of use, addiction liability, and mechanisms linking stress and cannabis use. For example, research suggests greater negative symptoms in females who use cannabis regularly (Lev-Ran et al., 2012). Females may also be particularly sensitive to the subjective effects of cannabis, which increases a female's vulnerability to dependence on this substance (Cooper and Haney, 2014). This is consistent with ample preclinical evidence indicating that females are more susceptible to all stages of substance use, from initiation to relapse (Anker and Carroll, 2011).

While there is a paucity of literature on the association between stress and cannabis use during pregnancy, the observations that do exist indicate that a variety of stressors (e.g., intimate partner violence, stressful life events, psychiatric co-morbidities) do, in fact, increase the risk of cannabis use during pregnancy. This is an unaddressed problem given *in-utero* exposure to cannabis leads to low birth weight, preterm birth, and other adverse pregnancy outcomes (Conner et al., 2016; Gunn et al., 2016; Campbell et al., 2018; Serino Ma et al., 2018; Brown et al., 2019b; Nashed et al., 2020; Sarrafpour et al., 2020). Moreover, there are currently no evidence-based interventions to reducing cannabis use during pregnancy. In fact, studies show that approximately half (48–50%) of healthcare providers in clinic and online visits did not respond to pregnant patients' reports of cannabis use (Holland et al., 2016; Brown et al., 2019a). Clearly, more research is needed to address this increasingly important public health issue.

## Proposed Framework

**Figure 1:**
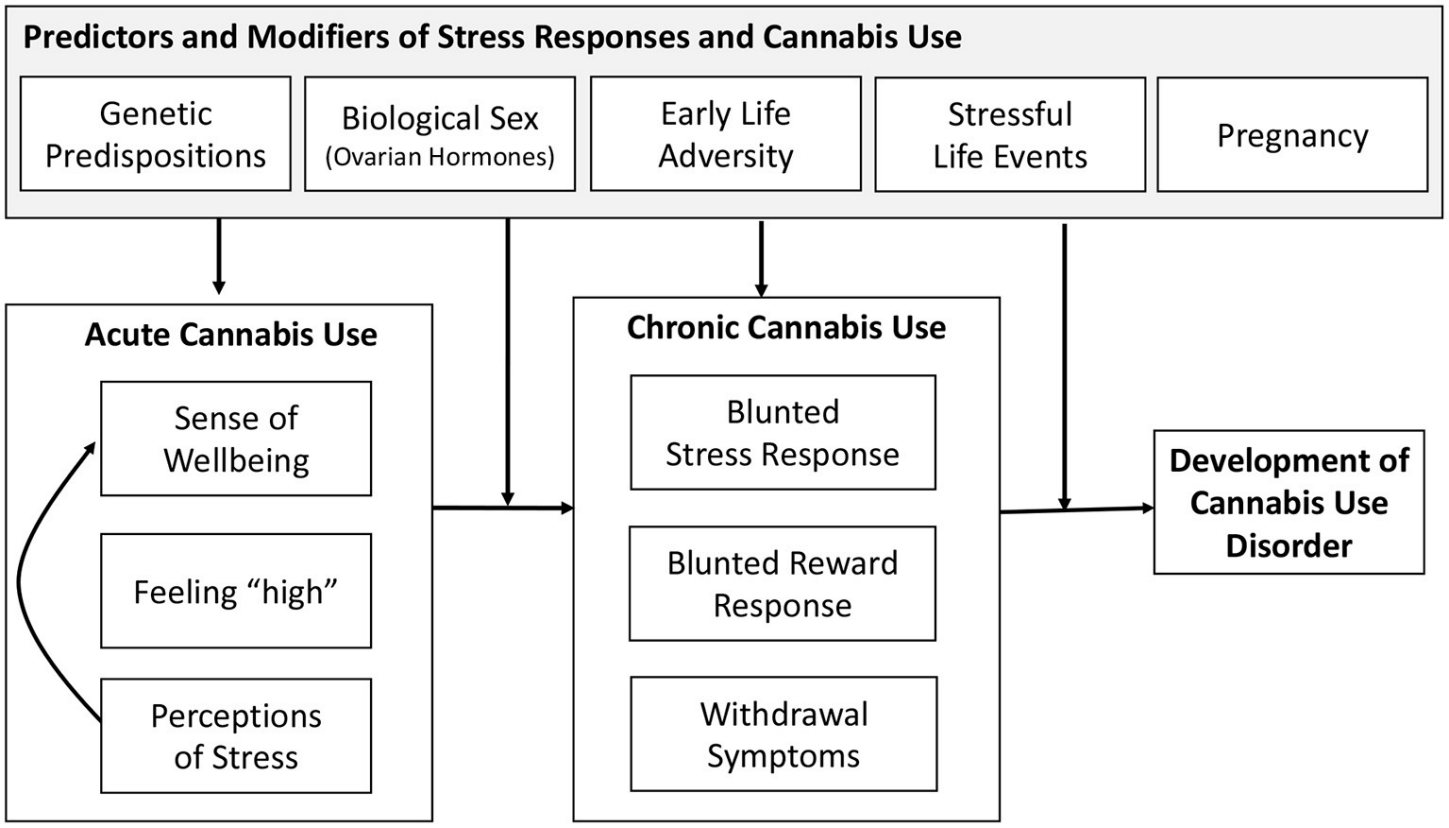
Framework of stress and development of cannabis use.

To aid in the future research in defining the nature of the bidirectional association between stress and cannabis use, we present a framework to guide future investigations in Figure 1. This framework indicates the acute effects of cannabis include an enhanced sense of well-being, feeling “high,” and reduced perceptions of stress. This leads to additional cannabis use via the desire to improve sense of well-being. These effects reinforce future cannabis use and contribute to maintained use through effects of chronic cannabis use. This reinforcement may be enhanced by a genetic predisposition or by exposure to stress, including early life adversity. Here, the role of stress is enhancing the reinforcing effects of cannabis use due to greater perceived relief from stress and negative affect that may result from cannabis use. This enhanced reinforcement could increase risk for physical dependence to cannabis and chronic cannabis use.

In light of the research reviewed above and the endocannabinoid (eCB) system in modulating the stress response as well as its interaction with the reward system (Volkow et al., 2017; Zehra et al., 2018), chronic exposure to cannabis and activation of the eCB system may lead to long-term neuronal adaptation processes and brain plasticity (Koob and Kreek, 2007; Koob and Le Moal, 2008). These long-term changes may be further pronounced in certain segments of the population, including those with history of early life adversity, those with genetic vulnerability, females, or during pregnancy; leading to further dysregulation of stress responses and contributing to alterations in brain reward systems. This, in turn, contributes to increases in stress perceptions and the need to treat those feelings with cannabis use. This escalation of use results in withdrawal symptoms during periods of abstinence, leading to maintenance and further escalation of cannabis use. It should also be noted that systems that are activated by stress and exhibit long-term changes by chronic cannabis use also show similar dysregulation associated with use of other substances (Tang et al., 2015; Koob and Volkow, 2016). Indeed, conditions associated with stress and anxiety have been reported to contribute to both cannabis and tobacco use (Hyman and Sinha, 2009; Feingold et al., 2016; Scherma et al., 2020), and like cannabis use, use of other substances has been associated with motivation to manage stress and negative affect (Hyman and Sinha, 2009; Farris et al., 2014; Cuttler et al., 2018).

## Future Directions

Examination of various parameters to capture the nature of stress response dysregulation under acute and chronic cannabis consumption remains a promising avenue for future research and intervention. This is indeed the case when considering the need to tease out changes in the stress response to the acute pharmacological effects vs. those that may be caused by withdrawal. The two distinct states (i.e., acute effects vs. withdrawal) engage different brain systems and are likely influenced by different environmental cues and individual difference factors. To that end, research is still needed to better understand threshold and mechanisms through which stress induces negative mood in individuals who use and do not use cannabis as well as to understand mechanisms that explain stress-elicited cravings for cannabis.

There are indications that eCB may have a buffering effect on stress, although eCB effects are influenced by dose, with low doses producing positive effects and high doses leading to negative effects. Defining parameters of how eCB may moderate the adverse effects of stress acutely and chronically is an important direction for future research, particularly in the context of how stress may increase vulnerability for using substances, such as cannabis. This also could stimulate research targeting stress-buffering therapeutic modalities mediated by this system.

More research is clearly warranted to elucidate sex differences (and gender differences) in cannabis use and stress-related HPA activity, as well as how these relationships are affected during pregnancy. The eCB system plays a crucial role in neuronal development and maturation of the brain, and, therefore, sex differences in brain maturation (De Bellis et al., 2001; De Bellis and Keshavan, 2003; Koolschijn and Crone, 2013; Nguyen et al., 2013) may influence the role of this system. It is possible that age at onset of cannabis use and sex additively influence stress patterns. However, this model has never been directly tested. Additionally, given the known variations in stress response systems during pregnancy, coupled with growing knowledge of the impact of cannabis use during pregnancy on adverse infant outcomes, additional research is needed to explore how further understanding of these relationships may offer critical information in the development of effective evidenced-based interventions to reduce perinatal cannabis use.

## Conclusions

Cannabis use, acutely and chronically, alters multiple stress response systems, and long-term use may lead to significant changes that promote maintenance of use, as well as dependence. Evidence to-date, while limited, suggests this may lead to an overall adverse effect on the stress response system. Research on the role of vulnerability factors (e.g., early life adversity), sex, and pregnancy, in the impact of cannabis use on stress and emotion regulation is lacking. Identification of the eCB system as a potential mechanism and regulator of the effects of stress and cannabis is important and has implications in the context of substance misuse and other stress-related disorders, including, posttraumatic stress disorder, depression, and other psychological disorders.

## Author Contributions

All authors listed have made a substantial, direct and intellectual contribution to the work, and approved it for publication.

## Conflict of Interest

The authors declare that the research was conducted in the absence of any commercial or financial relationships that could be construed as a potential conflict of interest.
